# Cloning and Characterization of a Flavonoid 3′-Hydroxylase Gene from Tea Plant (*Camellia sinensis*)

**DOI:** 10.3390/ijms17020261

**Published:** 2016-02-22

**Authors:** Tian-Shan Zhou, Rui Zhou, You-Ben Yu, Yao Xiao, Dong-Hua Li, Bin Xiao, Oliver Yu, Ya-Jun Yang

**Affiliations:** 1 Horticulture, Northwest A&F University, 3 Taicheng Road, Yangling 712100, China; zhoutianshan@nwsuaf.edu.cn (T.-S.Z.); yyben@163.com (Y.-B.Y.); xiaoyao1583@gmail.com (Y.X.); lidhua29@163.com (D.-H.L.); xiaobin@nwsuaf.edu.cn (B.X.); 2 Conagen Inc., 15 DeAngelo Dr., Bedford, MA 01730, USA; rui.zhou@conagen-inc.com; 3 Tea Research Institute, Chinese Academy of Agricultural Sciences, 9 Meiling South Road, Hangzhou 310008, China

**Keywords:** flavonoid 3′-hydroxylase, heterologous expression, flavan 3-ols, nitrogen depletion, *Camellia sinensis*

## Abstract

Tea leaves contain abundant flavan-3-ols, which include dihydroxylated and trihydroxylated catechins. Flavonoid 3′-hydroxylase (F3′H: EC 1.14.13.21) is one of the enzymes in the establishment of the hydroxylation pattern. A gene encoding F3′H, designated as *CsF3*′*H*, was isolated from *Camellia sinensis* with a homology-based cloning technique and deposited in the GenBank (GenBank ID: KT180309). Bioinformatic analysis revealed that *CsF3*′*H* was highly homologous with the characterized *F3*′*H*s from other plant species. Four conserved cytochrome P450-featured motifs and three F3′H-specific conserved motifs were discovered in the protein sequence of *CsF3*′*H.* Enzymatic analysis of the heterologously expressed *CsF3*′*H* in yeast demonstrated that tea F3′H catalyzed the 3′-hydroxylation of naringenin, dihydrokaempferol and kaempferol. Apparent *K*_m_ values for these substrates were 17.08, 143.64 and 68.06 μM, and their apparent *V*_max_ values were 0.98, 0.19 and 0.44 pM·min^−1^, respectively. Transcription level of *CsF3*′*H* in the new shoots, during tea seed germination was measured, along with that of other key genes for flavonoid biosynthesis using real-time PCR technique. The changes in 3′,4′-flavan-3-ols, 3′,4′,5′-flavan-3-ols and flavan-3-ols, were consistent with the expression level of *CsF3*′*H* and other related genes in the leaves. In the study of nitrogen supply for the tea plant growth, our results showed the expression level of *CsF3*′*H* and all other tested genes increased in response to nitrogen depletion after 12 days of treatment, in agreement with a corresponding increase in 3′,4′-catechins, 3′,4′,5′-catechins and flavan 3-ols content in the leaves. All these results suggest the importance of *CsF3*′*H* in the biosynthesis of 3′,4′-catechins, 3′,4′,5′-catechins and flavan 3-ols in tea leaves.

## 1. Introduction

**Figure 1:**
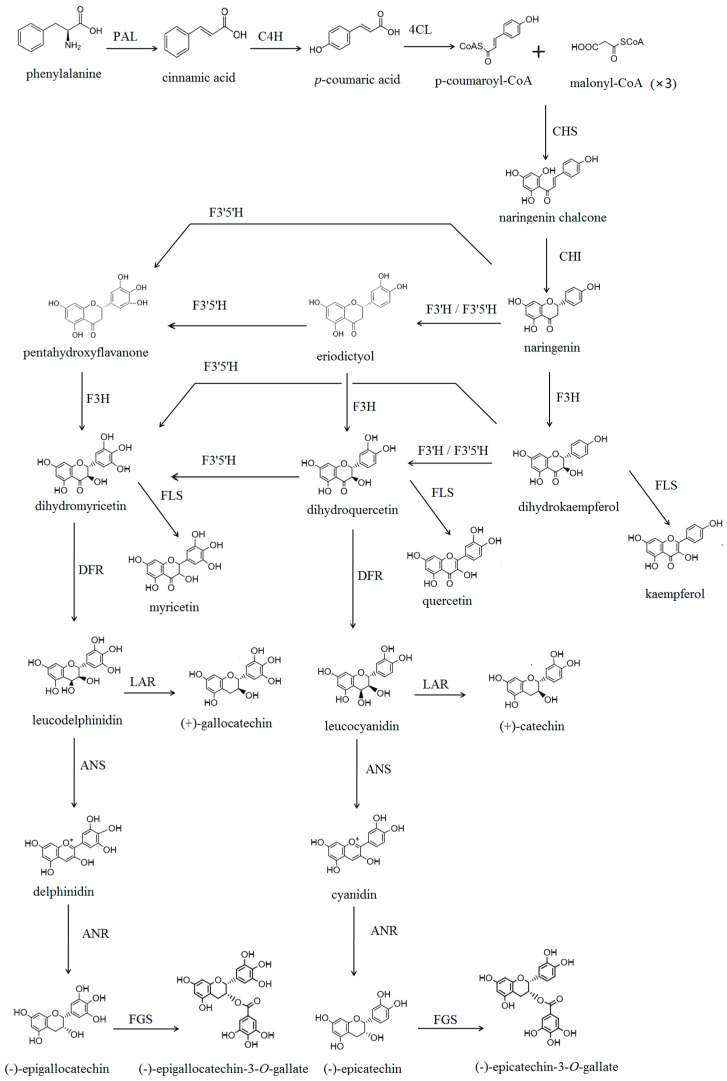
Suggested biosynthetic pathways of flavan 3-ols in *Camellia sinensis* leaves. Abbreviations of enzymes are as follows: PAL, phenylalanine ammonia-lyase (EC 4.3.1.24); C4H, cinnamic acid 4-hydroxylase (EC 1.14.13.11); 4CL, 4-coumarate-CoA ligase (EC 6.2.1.12); CHS, chalcone synthase (EC 2.3.1.74); CHI, chalcone isomerase (EC 5.5.1.6); F3H, flavanone 3-hydroxylase (EC 1.14.11.9); F3′,5′H, flavonoid 3′,5′-hydroxylase (EC 1.14.13.88); F3′H, flavonoid 3′-hydroxylase (EC 1.14.13.21); FLS, flavonol synthase (EC 1.14.11.23); DFR, dihydroflavanol 4-reductase (EC 1.1.1.219); ANS, anthocyanidin synthase (EC 1.14.11.19); ANR, anthocyanidin reductase (EC 1.3.1.77); LAR, leucocyanidin reductase (EC 1.17.1.3); FGS, flavan 3-ol gallate synthase (EC number not assigned).

Tea (*Camellia sinensis*) is an important economic crop native to southwestern China [1]. Tea leaves can be processed into the most widely consumed beverage. Increasing evidence suggests that tea extracts produce beneficial health effects, such as anticancer [2], anti-vascular disease [3], anti-bacterial [4], anti-inflammation [5], and anti-allergic [6] activities. The leaves of tea plants have large amounts of flavonoids, which include flavones, flavanones, flavonols, flavan 3-ols (as known as catechins), and anthocyanidins [7]. The major flavonoid compounds in tea are flavan 3-ols, which possess strong radical scavenging and antioxidant effects [8]. Flavan 3-ols are synthesized through the phenylpropanoid and flavonoid pathways [9,10,11,12], illustrated in Figure 1. The entry point for the biosynthesis of flavan 3-ol is the formation of chalcone with the catalyzation of chalcone synthase (CHS). The other enzymes involved in this biosynthetic pathway include chalcone isomerase (CHI), flavanone 3-hydroxylase (F3H), dihydroflavonol 4-reductase (DFR), leucocyanidin reductase (LAR), anthocyanidin synthase (ANS), anthocyanidin reductase (ANR), and flavan 3-ol gallate synthase (FGS, no report) [10,11].

Major flavan 3-ols in tea include (+)-catechin, (−)-epicatechin, (−)-epicatechin gallate, (−)-epigallocatechin (+)-gallocatechin and (−)-epigallocatechin gallate. Based on the hydroxylation position in the B-ring, catechins can be categorized in several subclasses, including 3′,4′-dihydroxylated catechins (3′,4′-flavan 3-ols) and 3′,4′,5′-trihydroxylated catechins (3′,4′,5′-flavan 3-ols). The ratio of dihydroxylated to trihydroxylated catechins (RDTC) in tea was used as an indicator of tea quality in the breeding programs [13,14,15]. The hydroxylation pattern of the B-ring of flavonoids was found to be determined by F3′H and flavonoid 3′,5′-hydroxylase (F3′5′H), which catalyze the hydroxylation at the 3′- or the 3′-and 5′-position of flavonoids, respectively [12,16,17,18]. F3′H and F3′5′H are classified to the subfamilies of CYP75B and CYP75A, respectively, in the superfamily of cytochrome P450-dependent monooxygenases [19]. In flavonoid biosynthesis, F3′H hydroxylates the B-ring of naringenin and dihydrokaempferol at the 3′-position to form eriodictyol and dihydroquercetin, respectively, the two important intermediates for the biosynthesis of 3′,4′-dihydroxyl flavonoid end-products formed in plants. Even though tea *F3*′*H*s were reported before [20], the function has not been fully characterized yet. The enzymatic characterization of CsF3′H is essential for us to understand the role of CsF3′H in the flavonoid biosynthetic system (Figure 1). Furthermore, the content of flavonoids in plant was influenced by environmental conditions such as nitrogen supply [21,22]. Study of the expression of *F3*′*H* and other genes related to flavonoid under limited nitrogen condition would help the functional characterization *in vivo*.

With a goal to functionally characterize CsF3′H *in vitro* and *in vivo*, we isolated a CsF3′H gene from tea by homologous cloning. Enzymatic analysis of the heterologous expressed *CsF3*′*H* in yeast revealed that naringenin is the optimal substrate for this enzyme. The expression pattern of *CsF3*′*H* correlated positively with 3′,4′-flavan 3-ols, 3′,4′,5′-flavan 3-ols and flavan 3-ols accumulation pattern in the tea seedling during the growth period. The expression level of *CsF3*′*H* also responded to nitrogen supplication, with the expression level of *CsF3*′*H* increased significantly after nitrogen depletion, resulting in an increased content of 3′,4′-catechins in the leaves.

## 2. Results and Discussion

### 2.1. Cloning and Sequence Analysis of CsF3′H Gene

A fragment of 1056 bp was obtained from PCR amplification of the partial target cDNA with degenerated primers. Subsequent 5′-RACE and 3′-RACE yielded an 1120 bp 5′-end and an 1447 bp 3′-end cDNA fragments, respectively. A full length cDNA sequence of flavonoid 3′-hydroxylase was then obtained by using the SeqMan program in DNAStar 7.1 (DNASTAR Inc., Madison, AL, USA). The full transcript has 1706 nucleotideswith a 5′-untranslated region (UTR) of 26 bp, an open reading frame (ORF) of 1557 bp and a 3′-UTR of 124 bp (Genbank ID: KT180309). *CsF3*′*H* was predicted to encode a protein with 518 amino acids, a theoretical molecular weight of 57.07 kDa and a calculated isoelectric point of 6.82.

**Figure 2:**
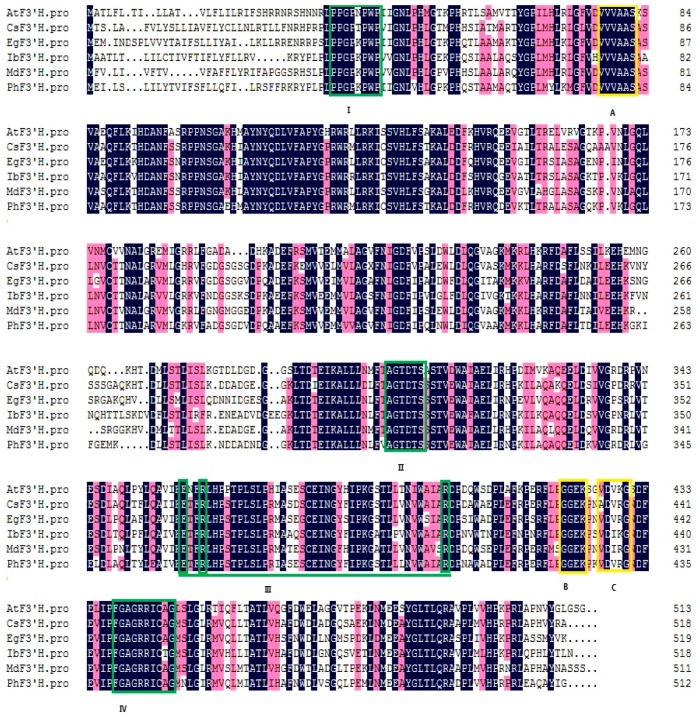
Multiple sequence alignment of the CsF3′H protein and five flavonoid 3′-hydroxylases (F3′Hs). Besides CsF3′H, other amino acid sequences included in this alignment were AtF3′H (Genbank ID: CAB62611.1); PhF3′H (Genbank ID: AAD56282.1); EgF3′H (Genbank ID: BAP94456.1); MdF3′H (Genbank ID: ACR14867.1) and IbF3′H (Genbank ID: AEH42499.1). At, *Arabidopsis thaliana*; Ph, *Petunia hybrida*; Eg, *Eustoma grandiflorum*; Md, *Malus×domestica*; Ib, *Ipomoea batatas*. Dark-blue shading and pinkish shading reflect 100% and 75% amino acid residues conservation, respectively. The P450-featured conserved motif, including the proline-rich “hinge” region (I), oxygen binding pocket motif (II), E-R-R motif (III) and heme-binding domain (IV) are boxed in green. Three F3′H-specific conserved motifs are boxed in yellow (marked A, B and C).

Four cytochrome P450-specific conserved motifs and three F3′H-specific conserved motifs were found in CsF3′H amino acid sequence using the Conserved Domain program at the NCBI website (showed in Figure 2). The proline-rich “hinge” region (P_36_PGPTPWP_43_) is supposed to be required for optimal orientation of P450 enzymes [23,24,25]. The heme domain (F_448_GAGRRICAG_457_) is responsible for the enzyme to bind carbon monoxide [19]. CsF3′H bears the motif A_312_GTDTS_315_, forming a binding pocket for oxygen molecule, which is required for its catalytic activity [26]. An E_369_-R_371_-R_411_ triad forming the pocket locking motif for the stabilization of the core structure [27], is also present in CsF3′H. In addition, two F3′H-specific motifs “VVVAAS” and “GGEK” [28] have high similarity counterparts at V_79_VVAAS_84_ and G_430_GEK_433_ in CsF3′H, respectively. But the third F3′H-specific motifs VDVKG [28] was present at A_436_DVRG_440_ of CsF3′H.

Five well characterized F3′Hs from other plant species, MdF3′H from *Malus × domestica* (GenBank ID: ACR14867.1) [29], PhF3′H from *Petunia hybrida* (GenBank ID: AAD56282.1) [17], EgF3′H from *Eustoma grandiflorum* (GenBank ID: BAP94456.1) [30], IbF3′H from *Ipomoea batatas* (GenBank ID: AEH42499.1) [31] and AtF3′H from *Arabidopsis thaliana* (GenBank ID: CAB62611.1) [18] were selected for sequence comparison. Multiple amino acid sequence alignment revealed high homology to these five F3′H sequences. *CsF3*′*H* showed 76%, 75%, 73%, 67% and 66% identities with them, respectively (Figure 2).

**Figure 3:**
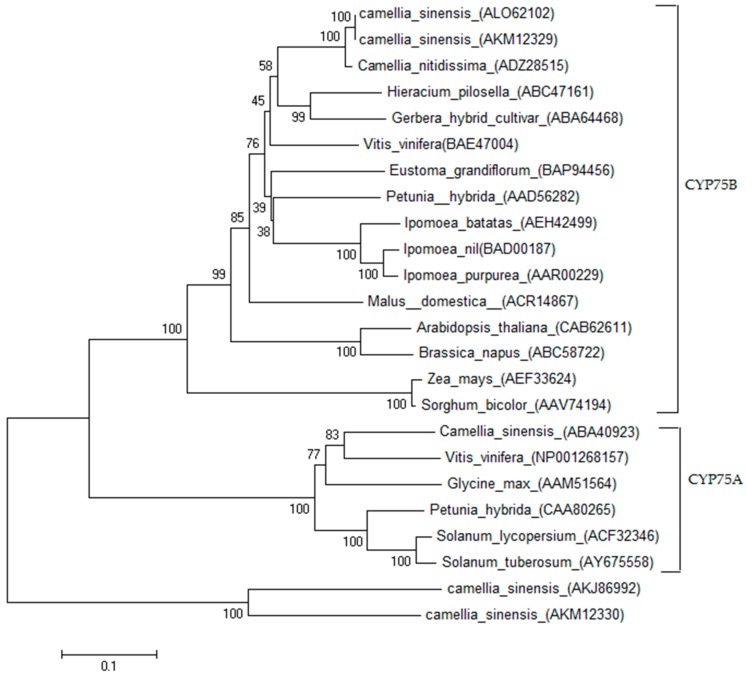
Phylogenetic analysis of CsF3′H protein and the proteins of F3′H and F3′5′H from other species. The phylogenetic tree was constructed from the ClustalW alignment using the neighbour joining method by the MEGA 6.0 program. Branches were labelled with the protein names and GenBank accession numbers. The scale bar represents 0.1 substitutions per site, and the numbers next to the nodes were bootstrap values from 1000 replicates.

A phylogenetic tree (Figure 3) was generated with several F3′H protein sequences from several plant species, including the reported tea F3′Hs [20] and F3′5′Hs. The tree clearly showed that F3′Hs and F3′5′Hs were clustered in the CYP75B and CYP75A clades, respectively. CsF3′H was grouped into the CYP75B subfamilies, and tightly related to the F3′H from *Camellia nitidissima.* In addition, CsF3′H and the CsF3′H2 (GenBank ID: AKM12329) [20] were clustered together. But two other CsF3′Hs (CsF3′H1, GenBank ID: AKJ86992 and CsF3′H3, GenBank ID: AKM12330) [20] were clustered neither in CYP75B nor in CYP75A.

### 2.2. Substrate Specificity of CsF3′H

**Figure 4:**
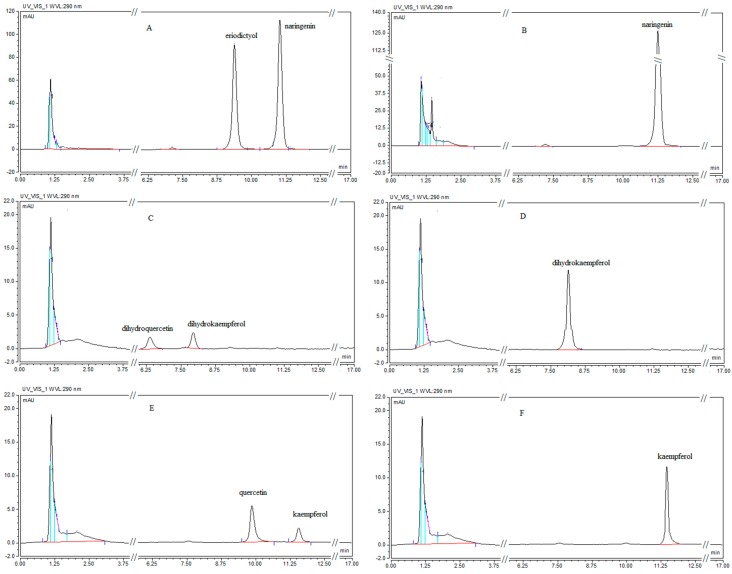
HPLC chromatograms of products from yeast cells with naringenin, dihydrokaempferol and kaempferol as substrates. HPLC chromatograms of products from pYES-dest52-*CsF3*′*H* with naringenin (**A**), dihydrokaempferol (**C**) and kaempferol (**E**) as substrates; HPLC chromatograms of products from the control with naringenin (**B**), dihydrokaempferol (**D**) and kaempferol (**F**) as substrates.

The yeast strain *Saccharomyces cerevisiae* WAT11, originally engineered to over-express a P450 reductase from *Arabidopsis thaliana* [32], was identified as a good heterologous host for plant P450 protein expression [12,16,33]. In the present study a vector of pYES-DEST52-*CsF3*′*H* was introduced into WAT11 and an empty pYES-DEST52 vector was transformed into WAT11 as a control. According to some previous findings [17,18,34,35], naringenin, dihydrokaempferol and kaempferol were chosen to assess the substrate specificity (Figure 4). WAT11 cells transformed with pYES-DEST52-*CsF3*′*H* vector catalyzed the hydroxylation at B-ring 3′-position of naringenin, dihydrokaempferol and kaempferol to eriodictyol, dihydroquercetin and quercetin, respectively, which indicates a broad substrate specificity of CsF3′H. In a recent report [36] leucopelargonidin was demonstrated to be also a substrate for F3′H. Due to the limited availability of the substrate, this compound was not covered in the present study.

Microsomal fractions from WAT11 cells harboring pYES-DEST52-*CsF3*′*H* were prepared and tested for NADPH-dependent 3′-hydroxylation of flavonoids using naringenin, dihydrokaempferol and kaempferol as substrates. The control microsomes did not show any activity. The apparent *K*_m_ values of F3′H for these flavonoids were measured to be 17.08, 143.64 and 68.06 μM, and their apparent *V*_max_ values were 0.98, 0.19 and 0.44 pM·min^−1^, respectively. (Table 1 and Figure S1). The *k*_cat_*/K*_m_ values indicated that naringenin is the preferred substrate for CsF3′H enzyme (Table 1).

It should be noted, however, that the substrates we used are racemic mixtures. F3′H was shown to be highly stereospecific for the 2*S*-enantiomer [37,38]. Currently we have no idea whether the presence of 2*R*-enantionmer disturbs the enzymatic reaction or not. As the enantiomeric pure flavonoid compounds were not commercially available, and the facilities required for purifying them were not available either, we cannot exclude the possibility that stereochemistry of the substrates (naringenin and dihydrokaempferol) used in our present study might influence the above results.

### 2.3. Gene Expression and Flavan 3-ol Accumulation in Tea Seed Germination

**Figure 5:**
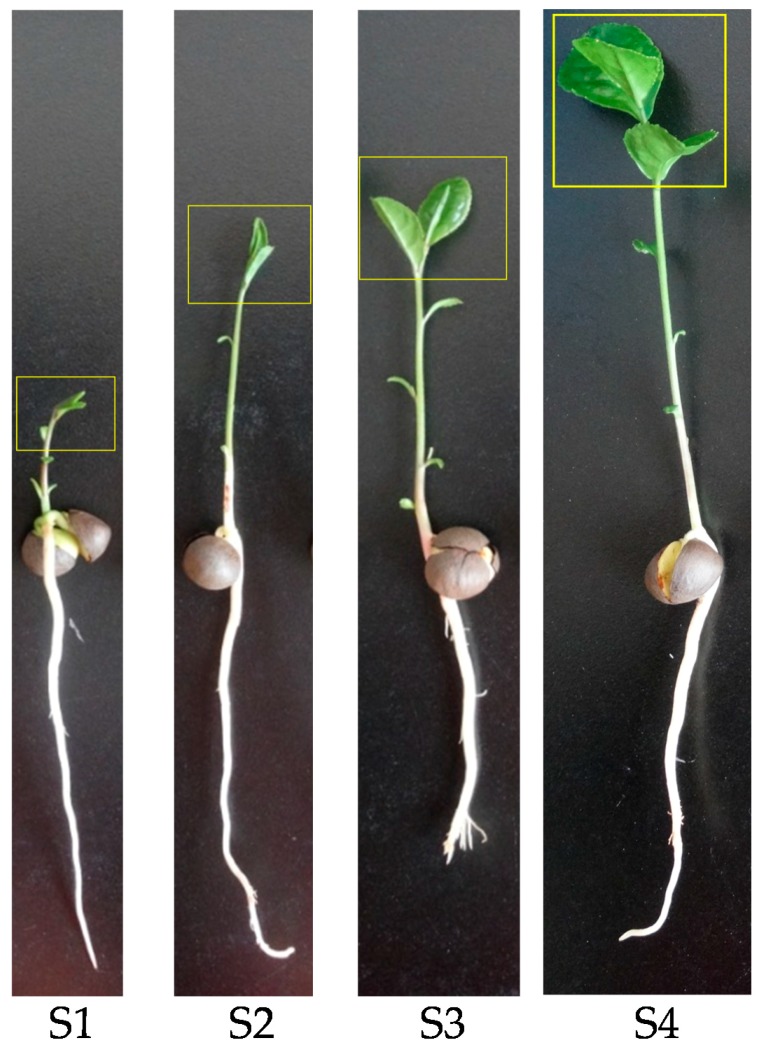
Tea seedlings at different developmental stages. **S1**, **S2**, **S3** and **S4** indicate the tea seedling at 20, 30, 40 and 50 d after germination respectively. Sampling sites were boxed in yellow.

The new shoots (including shoot apex and developing leaves) were collected at 20, 30, 40 and 50 days after tea seed germination respectively. As shown in Figure 5, the developmental process of tea seedling was divided into four stages, during which the new tea shoots gradually changed from one bud (S1) to one bud with three leaves (S4).

#### 2.3.1. Gene Expression

**Figure 6:**
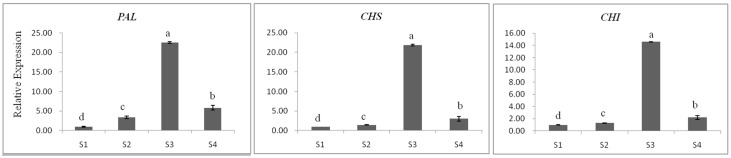

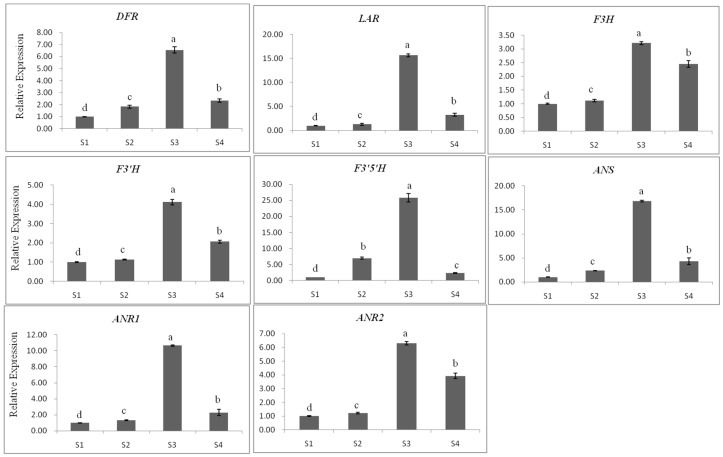
Expression levels of *CsF3*′*H* & other flavan 3-ol biosynthesis related genes for tea seedling at different stages. The data represent the mean ± SD from three independent measurements. Means in each column for each genes labeled with the same letter are not significantly different (*p* > 0.05) based on one-way ANOVA with Duncan’s multiple range test.

We compared the expression profiles of *CsF3*′*H* and other flavan 3-ol biosynthetic genes (*PAL*, *CHS*, *CHI*, *F3H*, *F3*′*5*′*H*, *DFR*, *LAR*, *ANS*, *ANR1* and *ANR2*) by quantitative (q)RT-PCR. The changes in the expression levels for flavan 3-ol biosynthesis related genes are shown in Figure 6. Interestingly, *CsF3*′*H* and other flavan 3-ol biosynthetic genes showed similar variation pattern during the plant growth. Their expression level gradually increased to the maximum by S3 and decreased rapidly in S4. But the increment varied among of them. Compared to S1, the highest expression level of *PAL*, *CHS*, *CHI*, *LAR*, *F3*′*5*′*H*, *ANS* and *ANR1* recorded at S3 were 22.51-, 21.85-, 14.6-, 15.64-, 25.82-, 16.85- and 10.66-fold respectively. However, the expression levels of *DFR*, *F3H*, *F3*′*H* and *ANR1* were high in S3, only 6.54-, 3.21-, 4.12- and 6.29-fold compare to S1 respectively.

#### 2.3.2. Flavan 3-ol Accumulation in Tea Seed Germination

**Figure 7:**
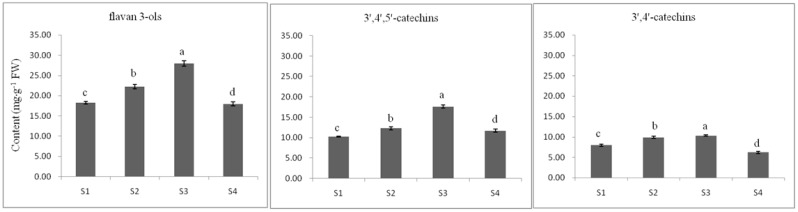

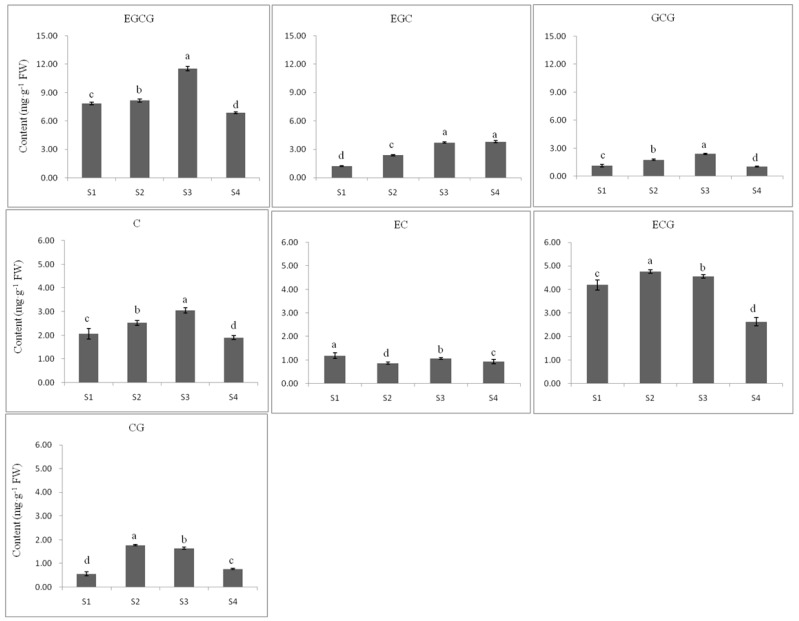
Content of flavan 3-ols in tea seedling at different developmental stages. The data represent the mean ± SD from three independent measurements. Means in each column for each flavan 3-ol labeled with the same letter are not significantly different (*p* > 0.05) based on one-way ANOVA with Duncan’s multiple range test.

The HPLC analysis demonstrated that the contents of seven typical tea flavan 3-ols in the leaves varied in different developmental stages (Figure 7). Among these flavan 3-ols, epigallocatechingallate (EGCG), which ranged from 6.89 mg·g^−1^ (S4) to 11.52 mg·g^−1^ (S3), was the most abundant, and catechin (C) was the least, ranging from 1.90 mg·g^−1^ (S4) to 3.06 mg·g^−1^ (S3). Based on the number of hydroxyl groups in the B-ring, flavan 3-ols can be classified into dihydroxylated catechins (3′,4′-catechins) and trihydroxylated catechins (3′,4′,5′-catechins). C, EC, ECG and CG are 3′,4′-catechins and EGC, EGCG and GCG are 3′,4′,5′-catechins. The concentration of 3′,4′-catechins changed from 6.23 mg·g^−1^ (S4) to 11.53 mg·g^−1^ (S1) and that of 3′,4′,5′-catechins from 10.25 mg·g^−1^ (S1) to 17.63 mg·g^−1^ (S3). It is obvious that the concentration of 3′,4′,5′-catechins were higher than that of 3′,4′-catechins in all stages, which is in agreement with previous reports [11,39]. Flavan 3-ol concentration obtained by summarizing all the individual components had a range from 17.96 mg·g^−1^ (in S4) to 27.96 mg·g^−1^ (in S3). During the process the changes in C, EGCG, GCG, 3′,4′-catechins, 3′,4′,5′-catechins and flavan 3-ols, showed similar patterns, with a gradual increase from S1 to S3, then decrease in S4. This variation trend was consistent with the expression level of the genes involved in the biosynthesis of flavan 3-ols.

#### 2.3.3. The Relationship between Gene Expression and Accumulation of Flavan 3-ols

The relationship between the expression of genes involved in flavonoid biosynthesis and accumulation of flavan 3-ols were analyzed (Table 2). The data indicated that the correlation coefficients between the expression levels of the key genes (*PAL*, *CHS*, *CHI*, *F3H*, *F3*′*5*′*H*, *DFR*, *ANS*, *LAR*, *ANR1* and *ANR2*) and the concentration of flavan 3-ols (or 3′,4′,5′-catechins) were higher than that of 3′,4′-catechins. *CsF3*′*H* showed similar pattern with these investigated genes with this issue in the present study, suggesting its important role in flavonoid biosynthesis.

### 2.4. Flavan 3-ols Accumulation and Gene Expression Response to Nitrogen Dificiency

**Figure 8:**
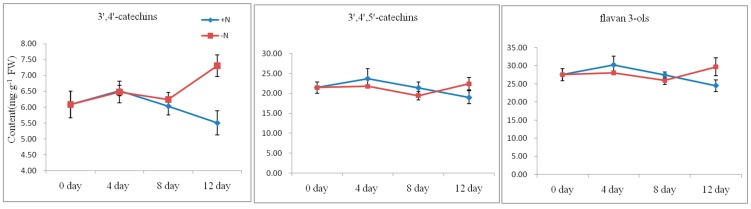
Content of flavan 3-ols in nitrogen deprived tea plants. The data represent the mean ± SD from three independent measurements.

Seven of the typical tea flavan 3-ols were detectable in both nitrogen replete plants and nitrogen deprived plants from 0 to 12 day. The concentration of 3′,4′-catechins (C, EC, ECG and CG), 3′,4′,5′-catechins (EGC, EGCG and GCG) and flavan 3-ols were compared and the results are shown in Figure 8. In the nitrogen replete plants, the concentration of 3′,4′-catechins ranged from 5.51 mg·g^−1^ (day 12) to 6.52 mg·g^−1^ (day 4) and that of 3′,4′,5′-catechins ranged from 19.02 mg·g^−1^ (day 12) to 23.74 mg·g^−1^ (day 4). Flavan 3-ols concentration ranged from 24.53 mg·g^−1^ (day 12) to 30.26 mg·g^−1^ (day 4). During the process the changes in 3′,4′-catechins, 3′,4′,5′-catechins and flavan 3-ols, showed similar patterns, which increased from day 0 to day 4 then gradually decreased from day 4 to day 12. However, in nitrogen deprived plants, the concentration of 3′,4′-catechins varied from 6.08 mg·g^−1^ (day 0) to 7.31 mg·g^−1^ (day 12) and that of 3′,4′,5′-catechins varied from 19.43 mg·g^−1^ (day 8) to 22.41 mg·g^−1^ (day 12). Flavan 3-ols concentration changed from 25.92 mg·g^−1^ (day 8) to 29.71 mg·g^−1^ (day 12). During nitrogen deficiency treatment the changes in 3′,4′-catechins, 3′,4′,5′-catechins and flavan 3-ols in the shoot tissues, increased from day 0 to day 4 then decreased from day 4 to day 8 and then increased from day 8 to day 12. The concentration of 3′,4′-catechins, 3′,4′,5′-catechins and flavan 3-ols on day 12 was 1.33-, 1.18- and 1.21-fold higher, respectively, in nitrogen deprived plants than in the plants given full nutrient solution. From day 8 to day 12, 3′,4′-catechins, 3′,4′,5′-catechins and flavan 3-ols showed an increasing trend in response to nitrogen deprivation.

**Figure 9:**
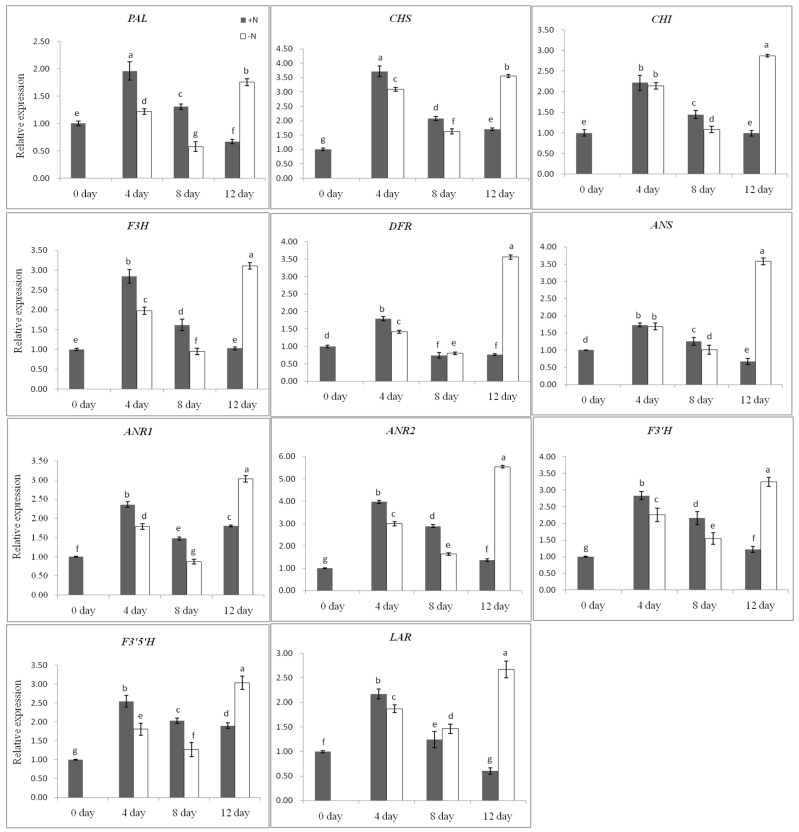
Expression levels of *CsF3′H* and other flavan 3-ol biosynthesis related genes in nitrogen deprived tea plants. The data represents the mean ± SD from three independent measurements. Means in each column for each genes labeled with the same letter are not significantly different (*p* > 0.05) based on one-way ANOVA with Duncan’s multiple range test.

The expression level of the gene was measured by real-time PCR, using the sample harvested on day 0 as calibrator (Figure 9). In the plant receiving nitrogen, expression of the *F3′H* gene and nine other key genes (except *ANR1*) have the same pattern, with lower expression on day 0, increased to a maximum by day 4, and then decreased over time, which were consistent with the expression profile in developmental stages (Figure 6). Whereas the plant depleted nitrogen, the expression of all genes tested showed similar features, with low expression on day 0, increased by day 4 and decreased by day 8, and then increased to a maximum by day 12. The expression of *F3′H* and ten other genes (*PAL*, *CHS*, *CHI*, *F3H*, *F3′5′H*, *DFR*, *LAR*, *ANS*, *ANR1* and *ANR2*) on day 12 was 2.61-, 2.59-, 2.03-, 2.83-, 2.98-, 1.61-, 4.66-, 4.31-, 5.33-, 1.66- and 4.02-fold higher, respectively, in the plants deprived of nitrogen as compared to that in the plants given full nutrient solution. All genes showed a general increase in their expressions in tea plants in response to nitrogen deprivation from day 8 to day 12 (Figure 9).

The increase in flavan 3-ol biosynthesis-related genes, and the increase in flavan 3-ols content during nitrogen deficiency from day 8 to day 12 are consistent. However, these increases were lower than those in several other plants such as *Arabidopsis thaliana* [40,41], and tomato [42,43]. In addition, it took a longer time for tea plants to increase flavonoid synthesis and the expression level of related-genes in response to nitrogen deficiency. The reason for this needs to be further investigated in the future.

## 3. Experimental Section

### 3.1. Plant Materials

Samples of *Camellia sinensis* cv. Wuniuzao, were obtained from Xixiang Tea Experimental Station of Northwest A&F University at Xixiang (Hanzhong, China), which is situated at 32°58′N and 107°40′E and 450 m above sea level. The new shoots (one leaf and one bud) were plucked and immediately frozen in liquid nitrogen, and stored at −80 °C until use in the study.

To detect the expression of genes during tea seed germination, mature fruits were collected from tea plant of *Camellia sinensis* cv. Wuniuzao growing in Xixiang Tea Experimental Station of Northwest A&F University. The seeds were sorted in water for 2 to 3 h after the fruit coat was removed. Only the seeds that sunk to the bottom of water were selected and sown in a soil mix (grass charcoal/perlite (3/1, *v*/*v*)) in plastic pots, which were kept at 25 °C under a 12/12 h (day/night) photoperiod for seed germination. The light was provided by cool-fluorescent tubes at a photon flux density of 52 μmol·m^−2^·s^−1^. The shoot tops (including shoot apex and developing leaves) were collected at 20, 30, 40 and 50 days after germination respectively, immediately frozen in liquid nitrogen and stored at −80 °C until analysis. The frozen tissues were pulverized in liquid nitrogen. RNA and flavonoid analysis were performed with the same powder. The samples were taken from three to eight plants.

To explore the effect of nitrogen depletion on the expression of genes, one-year-old rooted cuttings of tea plant (*Camellia sinensis* cv. Wuniuzao) were employed as the experimental materials. After washing the roots thoroughly, the cutting plants were transplanted in plastic pots containing hydroponic culture nutrient solution [44]. The basal nutrient solution was supplied stepwise at 1/5 strength of its concentration for 7 days, 1/2 strength for 7 days, and full strength for the other days before treatment. The pH of the solution was adjusted to 4.5 every two days with 5 mol·L^−1^ H_2_SO_4_. All the culture solutions were renewed once a week. During the culture of plants, forced aeration was performed. All cultures were maintained in the same conditions for seed germination described above. When the plants was putting out new shoots (one bud and two leaves), the plants were shifted to a nitrogen deprived regimen where (NH_4_)_2_SO_4_ was changed to K_2_SO_4_ and Ca(NO_3_)_2_·4H_2_O was changed to CaCl_2_·2H_2_O.

The roots of tea plants were washed in dilute H_2_SO_4_ (pH 3.0) and rinsed with distilled water to remove nutrients (referred to as day 0). Then tea plants were divided into two groups randomly. One group was shifted to deprived nitrogen solution and another group was shifted to complete solution. Samples (one bud and two leaves) were harvested and pooled from three plants with nitrogen supply and three plants without nitrogen supply at day 4, day 8 and day 12 respectively. The tissues were harvested, frozen, stored and ground to powder for analysis as described above. The samples for RNA and flavonoid analysis were taken from the same powder.

### 3.2. Isolation of the Flavonoid 3′-Hydroxylase Gene

Total RNA was extracted from the leaves of *Camellia sinensis* cv. Wuniuzao using RNAiso Plus Total RNA kit (TaKaRa, Japan). Single-strand cDNA was synthesized from 5 μg of total RNA with an oligo(dT)17 primer using PrimeScript TM RT reagent kit (TaKaRa, Japan) according to the manufacturer’s protocols. The resultant single-strand cDNA was used as the template for PCR amplification of the target cDNA with 1.5 units Phusion^®^ High-Fidelity DNA polymerase (New England Biolabs, Boston, MA, USA) and degenerated primers (forward, CACCMGNCCNCCNAAYWSNG-GNGCC; reverse, CCNTTYGGNGCNGGNMGNMGNATHTG) designed with reference to the conserved region of F3′H sequence from other plants. The PCR products were separated by agarose gel electrophoresis and purified with a QIAEX II Gel Extraction Kit (QIAGEN, Mansfield, MA, USA). The purified PCR fragments were then subcloned into a pENTR^TM^ TOPO^®^ vector (Invitrogen, Carlsbad, CA, USA) for sequencing.

To isolate a full-length cDNA fragment by RACE, specific primers were subsequently designed based on the core fragment of *CsF3′H*. The 3′-end and 5′-end of *CsF3*′*H* cDNA were amplified with the SMART™ RACE cDNA Amplification Kit (Clontech, Mountain View, CA, USA). The 5′ forward primers was 5′GSP (AGTCGAAAGGTTTCCTTGATGATGGC) and the 3′ reverse primer was 3′GSP (CGGCCACCCAACTCCGGTGCCAAAC). 5′ and 3′ RACE-PCR techniques were performed, using these gene specific primers and UPM (Universal Primer a mix, provided by the kit). The thermal cycling conditions were 94 °C for 5 min; followed by 35 cycles of 95 °C for 30 s, 60 °C for 30 s, and 72 °C for 1 min; and a final elongation step at 72 °C for 10 min. The purified 3′-RACE and 5′-RACE products were subcloned into pMD 18-T vectors for sequencing.

The full length cDNA sequence of flavonoid 3′-hydroxylase was analyzed with SeqMan program in DNAStar 7.1 (DNASTAR Inc., Madison, WI, USA). The ORF finder programmer at the NCBI website was used to search the open reading frames in the F3′H nucleotide sequences. Primers *CsF3*′*H* ORF forward (CACCATGACTTCCTTAGCTTTTGTTC) and *CsF3*′*H* ORF reverse (TTAGGCCCGATACACATGGGGTG) were used to amplify the ORF of *CsF3*′*H* with Phusion^®^ High-Fidelity DNA polymerase. PCR program was as follows: 98 °C for 3 min; followed by 35 cycles of 98 °C for 20 s, 58 °C for 30 s, and 68 °C for 1 min; and a final elongation step at 68 °C for 10 min before cooling to 4 °C. The product was ligated into a pENTR™ TOPO^®^ vector (Invitrogen, Carlsbad, CA, USA).

### 3.3. Bioinformatic Analysis

According to the open reading frames, the theoretical molecular weight and isoelectronic point of CsF3′H were calculated with ExPASy Compute PI/MW tool. Multiple alignments of protein sequences were performed by the Clustal W. The phylogenetic tree was constructed with the MEGA v5.2 software [45]. In MEGA, distance matrices were generated by the pairwise deletion option with the Poisson correction amino acid matrix. One thursand bootstrap replicates were made and trees were generated using neighbor-joining (NJ) method for each replicate. Percentage of replicates supporting each branch was given next to the nodes.

### 3.4. Yeast Expression and Microsome Preparation

The *CsF3′H* ORF was cloned into the destination vector pYES-dest52 from the entry vector pENTR-*CsF3′H* using Gateway LR Clonase enzyme (Invitrogen, Carlsbad, CA, USA). The obtained pYES-dest52-*CsF3′H* was introduced into *Saccharomyces cerevisiae* WAT11 with a transformatin kit (Frozon-EZ yeast Transformation II, Zymo Research, Irvine, CA, USA). Meanwhile, the empty vector pYES-dest52 was transformed as control.

A single colony from a SD-U medium plate was inoculated in 50 mL SD-U liquid medium containing 20 g·L^−1^ glucose, and the yeast cells were grown at 30 °C for 12 h. The cells were spun down (1500× *g*, 5 min) and resuspended in SD-U medium containing 20 g·L^−1^ galactose, and the resuspension was diluted to OD_600_ to 0.4 for substrate specificity assessment at 16 °C for 12 h. Naringenin, dihydrokaempferol and kaempferol solutions were fed into the culture to a final concentration of 5 mM, respectively. The reactions were stopped by sonication for 15 min after 12 h incubation. The products from each reaction were extracted with 10 mL ethyl acetate for three times, and organic extracts were pooled, evaporated and redissolved in 15 mL methanol for HPLC analysis.

For microsome induction, yeast cells were firstly propagated in SD-U liquid medium containing 20 g·L^−1^ glucose, then spun down (1500× *g*, 5 min) and resuspended in SD-U medium containing 20 g·L^−1^ galactose diluting the OD_600_ to 0.4. After induction at 16 °C for 24 h, the yeast microsomal fraction was prepared with MgCl_2_ according to Olsen *et al.* [16]. The resultant microsome was dissolved in pre-cooled 1.0 to 1.5 mL TEG (30% glycerol in 50 mM Tris–HCl with 1 mM EDTA) on ice. Protein concentrations of enzyme extract were spectrometrically determined using Coomassie Brilliant Blue G-250.

### 3.5. Enzyme Assays

As potential substrates fo CsF3′H, naringenin, dihydrokaempferol and kaempferol were tested with microsomes prepared from yeast pYES-dest52-*CsF3′H* transformants. The reaction solution contains 100 mM sodium phosphate buffer, pH 7.0 with 1.0 mM NADPH. The reaction was started by the addition of microsomes after the assay mixture was equilibrated for 15 min at 30 °C. The concentration of substrate in the assays was adjusted in the range of 1 to 300 μM. Total volume of the reaction system was 200 μL. After 30 min incubation the reaction was terminated by adding ethyl acetate. The products in each reaction were extracted and analyzed as described above. To validate that CsF3′H catalyzed the hydroxylations, control assays were run with microsomes prepared from WAT11 transformed with the pYES-dest52 vector without *CsF3′H* insertions.

### 3.6. HPLC Analysis

#### 3.6.1. Flavonoid Standards

(±)-Naringenin, (±)-eridodictyol, (±)-dihydrokaempferol, dihydroquercetin, kaempferol and quercetin were purchased from Sigma–Aldrich (USA). (+)-Catechin, (−)-epicatechin, (−)-epigallocatechin, (+)-gallocatechin, (−)-epicatechin 3-*O*-gallate and (−)-epigallocatechin 3-*O*-gallate were purchased from Shanghai RongHe Phar-maceutical Co. (Shanghai, China).

#### 3.6.2. Analysis of Enzyme Substrates and Products

The flavonoids were analyzed with a HPLC system (LC 20AD, Shimadzu Corporation, Japan) equipped with a Wondasil C18 column (Gl Sciences Inc., Torrance, CA, USA) and a diode array detector (SPD M20A, Shimadzu Corporation). The HPLC conditions were as follows: 10%–40% for 10 min, 40%–60% for 5 min and 60%–10% for 2 min at a flow rate of 1.0 mL·min^−1^ (the percentage represents the fraction of acetonitrile in the solution). The detection wavelength was set 290 nm. Injection volume was 10 μL and the separation temperature was set at 25 °C. Flavonoids generated from enzyme reactions were identified according to the retention time, UV-absorbance spectrum, and co-chromatography with authentic chemicals.

#### 3.6.3. Analysis of Flavan 3-ols in Leaves

The samples for analysis of flavan 3-ols were prepared as follows: 100 mg fresh leaves was grounded in liquid nitrogen and extracted with 1 mL 50% methanol by sonication at room temperature for 10 min. The mixture was centrifuged at 4000× *g* for 15 min and the resultant supernatant and residues were separated. The residues were re-extracted twice as above. All the supernatants were pooled and filtered through a 0.22 μm membrane for HPLC analysis.

The HPLC system and column were as described as above. The mobile phase used has 2% acetic acid in water (A) and acetonitrile (B), respectively. The elution gradient increased linearly from 6.5% to 25% B at 16 min, reduced back to 6.5% B at 25 min with a flow rate of 1.0 mL·min^−1^. Samples injection amount was 10 μL and the separation temperature was maintained at 30 °C. The detection wavelength was set 280 nm. One biological sample pooled from three individual plants was analyzed. Three analytical replicates were carried out for each sample.

### 3.7. Expression of Genes Related to Flavan 3-ol Biosynthesis by Real-Time PCR

Total RNA extraction and the first strand cDNA synthesis were carried out as described above. Real-time PCR reactions were assayed with an iQ5 Real-Time PCR System (Bio-Rad, Hercules, CA, USA) using SYBR^®^ Premix Ex Taq™ II (TaKaRa, Bio Inc., China) for detection. The PCR mixture contained 2.0 μL diluted cDNA (50 ng·μL^−1^), 10 μL SYBR^®^ Premix Ex Taq™ II, 0.8 μL of forward and reverse primers (10 μmol·L^−1^) in a final volume of 20 μL. The amplification was carried out with the following cycling parameters: 95 °C for 2 min, followed by 40 cycles at 95 °C for 30 s, 52 °C for 30 s, and 72 °C for 30 s. The primers for Real-Time RT-PCR are listed in Table 3. Data are expressed as mean value of three replicates, normalized against the expression levels of β-actin (HQ420251.1). The relative expression was calculated by Pfaffl’s method [46].

## 4. Conclusions

The *CsF3′H* gene was cloned and functionally characterized in our study. Bioinformatic analysis suggested that the *CsF3′H* was highly homologous with the characterized *F3′Hs* from other plant species. Heterologous expression of *CsF3′H* in yeast demonstrated that CsF3′H accepted naringenin, dihydrokaempferol and kaempferol as substrates, among which naringenin was shown to be the optimal substrate. During tea seed germination, the expression levels of *CsF3*′*H* correlated positively with 3′,4′-catechins, 3′,4′,5′-catechins and flavan 3-ol accumulation pattern in leaves. Expression of *CsF3*′*H* and all other tested genes in the flavonoid biosynthetic pathway increased in response to nitrogen deprivation, which were consistent with a corresponding elevation of 3′,4′-catechins, 3′,4′,5′-catechins and flavan 3-ols content. These results strongly suggest the importance of our cloned *CsF3*′*H* in the accumulation of the flavonoids in the tea leaves.

## Figures and Tables

**Table 1:** Steady-state kinetic parameters for CsF3′H in microsomes.

Substrate	Naringenin	Dihydrokaempferol	Kaempferol
*K*_m_ (μM)	17.08 ± 0.46	143.64 ± 0.69	68.06 ± 0.43
*V*_max_ (pM·min^−1^)	0.98 ± 0.01	0.19 ± 0.01	0.44 ± 0.01
*k_cat_* (pM·min^−1^·mg^−1^·microsome)	49.09 ± 0.21	9.86 ± 0.13	21.88 ± 0.35
*k_cat_* / *K*_m_ (×10^−3^ min^−1^·mg^−1^·microsome)	2.88 ± 0.07	0.07 ± 0.01	0.32 ± 0.01

The data represent the mean ± SD from three independent measurements.

**Table 2:** The correlation coefficiency between gene expression level and flavan 3-ols.

Correlation Coefficient	3′,4′-Catechins	3′,4′,5′-Catechins	Flavan 3-ols
*PAL*	0.513	0.981 *	0.885
*CHS*	0.552	0.970 *	0.893
*CHI*	0.557	0.970 *	0.895
*F3H*	0.077	0.812	0.591
*DFR*	0.526	0.986 *	0.893
*ANS*	0.508	0.977 *	0.879
*ANR1*	0.531	0.971 *	0.885
*ANR2*	0.166	0.861	0.661
*LAR*	0.506	0.967 *	0.872
*F3*′*H*	0.364	0.941	0.796
*F3*′*5*′*H*	0.723	0.990 *	0.975

* Significant correlation (*p* < 0.01).

**Table 3:** Primers for Real time PCR.

Gene	Accession No.	Forward Primer (5′–3′)	Reverse Primer (5′–3′)	Product Length (bp)
*PAL*	D26596	TCCAATTCCTTGCCAATCC	AACTGCCTCGGCTGTCTTTC	106
*CHS*	AY169403	ACAAAGGCAATCAAAGAATGG	ATGGGCGAAGACCGAGTAG	124
*CHI*	DQ904329	TGAGACTGAACCCAAGACCG	TAGATTTTGATGCCGATGCC	114
*F3H*	AY641730	TACCATCACCCTGCTCCTCC	CATTCTTGAACCTCCCATTGC	153
*F3′H*	KT180309	TCGACCAGAACGATTCCTACC	ACTGGACCATACGCAACCCTA	134
*F3′5′H*	DQ194358	TCTCAATCTTCCCAGAGTCGC	CAGTCTTCGCATTCTTTCCAC	173
*DFR*	AB018685	ATTCCCACCAAGCCTAATCAC	CCTGAGGACGCTCATACAAGA	137
*ANS*	AY830416	TTCAAGGGTATGGGAGCAAA	TGCAGGAATGTAGTCGGTTG	139
*LAR*	GU992401	AACTCACCCTAGTCCATGCCA	CACCCTCCTCTTTTCGTTGTA	134
*ANR1*	GU992402	CATAGCCGGTTGTGACCTTG	TGACACGTTTAACCGTTCCTG	147
*ANR2*	GU992400	CGAGACCCAGGCAATCAGA	ACCAGGTCACAACCCGCTA	131
*β-actin*	HQ420251.1	GCCATCTTTG ATTGGAATGG	GGTGCCACAACCTTGATCTT	175

## Supplementary Materials

Supplementary materials can be found at http://www.mdpi.com/1422-0067/17/2/261/s1.
