# Supplementary material for: Cloning and Characterization of a Flavonoid 3′-Hydroxylase Gene from Tea Plant (Camellia sinensis)
Source: Int J Mol Sci. 2016 Feb 22;17(2):261. doi: 10.3390/ijms17020261 (PMC4783990; doi:10.3390/ijms17020261)
Supplement: Supplementary file 1 [file ijms-17-00261-s001.pdf]

Supplementary Materials: Cloning and Characterization of a Flavonoid 3′-Hydroxylase Gene from Tea Plant (*Camellia sinensis*)

Tian-Shan Zhou, Rui Zhou, You-Ben Yu, Yao Xiao, Dong-Hua Li, Bin Xiao, Oliver Yu and Ya-Jun Yang

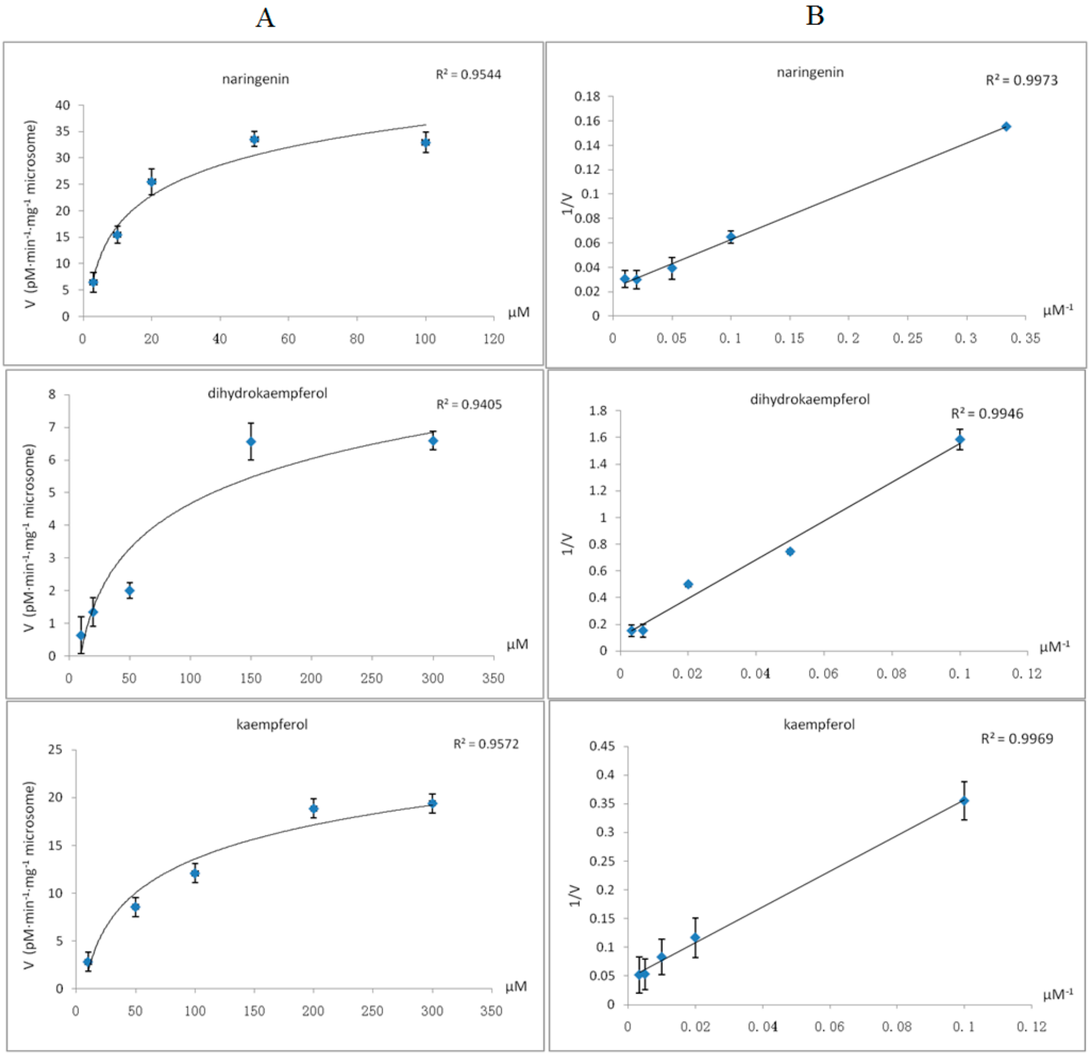

Figure S1. Concentration dependence of F3′H observed in yeast microsomes. F3′H-containing microsomes originating from transformed pYES-dest52-*CsF3′H* cells were incubated in 100 mM phosphate buffer pH 7.0 at 30 °C with naringenin, dihydrokaempferol and kaempferol as substrate respectively. (**A**) The result of a multi-iterative fitting of experimental data using the Michaelis-Menten equation; (**B**) Michaelis–Menten double-reciprocal plot. The data represent the mean ± SD from three independent measurements.
